# Exploring the characteristics of conversational agents in chronic disease management interventions: A scoping review

**DOI:** 10.1177/20552076241277693

**Published:** 2024-10-29

**Authors:** Ekaterina Uetova, Lucy Hederman, Robert Ross, Dympna O’Sullivan

**Affiliations:** 1 8819 School of Computer Science, Technological University Dublin, Dublin, Ireland; 2 8809 School of Computer Science and Statistics, Trinity College Dublin, Dublin, Ireland

**Keywords:** Conversational agent, chatbot‌, chronic disease, dialogue, ‌mobile health

## Abstract

**Objective:**

With the increasing global burden of chronic diseases, there is the potential for conversational agents (CAs) to assist people in actively managing their conditions. This paper reviews different types of CAs used for chronic condition management, delving into their characteristics and the chosen study designs. This paper also discusses the potential of these CAs to enhance the health and well-being of people with chronic conditions.

**Methods:**

A search was performed in February 2023 on PubMed, ACM Digital Library, Scopus, and IEEE Xplore. Studies were included if they focused on chronic disease management or prevention and if systems were evaluated on target user groups.

**Results:**

The 42 selected studies explored diverse types of CAs across 11 health conditions. Personalization varied, with 25 CAs not adapting message content, while others incorporated user characteristics and real-time context. Only 12 studies used medical records in conjunction with CAs for conditions like diabetes, mental health, cardiovascular issues, and cancer. Despite measurement method variations, the studies predominantly emphasized improved health outcomes and positive user attitudes toward CAs.

**Conclusions:**

The results underscore the need for CAs to adapt to evolving patient needs, customize interventions, and incorporate human support and medical records for more effective care. It also highlights the potential of CAs to play a more active role in helping individuals manage their conditions and notes the value of linguistic data generated during user interactions. The analysis acknowledges its limitations and encourages further research into the use and potential of CAs in disease-specific contexts.

## Introduction

In recent years, there has been a significant rise in the prevalence of chronic diseases, resulting in a growing number of individuals living with at least one chronic health condition.^1^ Chronic conditions have enduring and persistent effects, necessitating patients and healthcare professionals to navigate complex lifestyle and behavioral adjustments and engage in long-term management.^2^ Moreover, these conditions reduce quality of life and life expectancy and can escalate personal healthcare expenses due to disability, frequent hospitalizations, and the need for multiple treatment procedures.

In healthcare, the continuum of care^3^ is the concept of an integrated care system that guides and tracks patients over time through every subsequent step of health services. The care system contains a range of healthcare and social services, from preventive care to acute treatment, rehabilitation, and long-term care. Over time, the patient goes through various parts of the system, depending on the stage of the treatment or the need for care. The continuum of care aims to provide patients with the right care at the right time and in the most appropriate setting (e.g., home, rehabilitation centers, or hospitals), promoting better health outcomes and patient satisfaction.

Alongside work performed by healthcare professionals, there are often health-related activities that patients and their informal caregivers undertake in managing their health conditions, sometimes referred to patient work.^4^ The activities include various processes, from cognitive to physical, performed individually or collaboratively with others, including family and community members, and can be classified as visible when they are acknowledged and valued by others or invisible when they are taken for granted by others and consequently undervalued.^5^

Digital technologies have the potential to empower people, giving them a sense of agency and control, allowing them to extend their skills and knowledge and giving them access to experiences and functions that people did not have before.^6^ Technologies can reduce the burden of patient work or self-management of health conditions and serve as accessible alternatives to in-person support and supervision. One such technology is conversational agents (CAs), computer systems that imitate human-like conversations using natural language user interfaces involving images, text, and voice.^7^ CAs potentially offer the advantages of scalability, reduced costs, lowered stigma, and personalized health support available at any time.^8,9^ CAs can be delivered through text or speech, making them versatile for different target groups, including children and older individuals. CAs can address various healthcare needs, such as mental health management support,^10^ aid in chronic disease self-management,^11^ and lifestyle change facilitation, e.g., physical activity and dietary modifications.^12^

However, existing digital technologies designed for patients with chronic conditions face challenges adapting to changing health needs and goals. Specific subgroups significantly differ in healthcare preferences and goals and require different information and recommendations.^13^ Moreover, patients evolve and change the apps they use throughout the trajectory of their condition, from diagnosis to long-term care, as their health goals change over the course of their disease.^13^ The chronic illness trajectory model^14^ describes how the course of illness varies for each patient and changes over time. Patients may shift between different illness phases repeatedly in unpredictable and inconsistent ways, as their conditions fluctuate.

Digital systems that are not only focused on one specific stage of the disease but also adapt to the changing needs of users can enable sustained app usage by reducing the necessity to search for more appropriate apps. Such systems would facilitate continuous tracking of healthcare data over time, supporting individuals in effectively managing their health and reducing the disruption caused by switching between different apps.

Despite the extensive research on the application of CAs in healthcare, to our knowledge, there has been no scoping review of different types of CAs in chronic disease management with no constraints on demographics. Existing reviews often restricted their focus to specific health areas, such as mental health,^15–17^ smoking cessation,^18^ physical activity^19^ or body weight management^20,21^; population, e.g., young (25 years and younger),^17^ or adults (18 years and older)^22^; or agent types, e.g., embodied,^23,24^ voice-based,^25^ artificial intelligence-based^2,21,26,27^ CAs or CAs with unconstrained natural language input,^28^ e.g., free text or speech. Other reviews report solely on the evaluation outcomes, e.g., effectiveness and acceptability,^18,27,29^ or on one of the CAs’ aspects, e.g., personalization^30^ or design features.^24^

This paper provides the results of a literature analysis aimed at addressing several research questions related to the use of CAs in the context of chronic disease management. The research questions explored in this article are:

1. What are the health domains, and the characteristics of the end users, targeted for CA interventions?
2. What are the characteristics of the CA studies?
3. Do CAs address continuum of care and patient work concepts for self-management? Do CAs adapt to the changing needs of users?
4. What are the CAs’ characteristics? How do different types of CAs map with patients’ profiles and health domains?

By addressing these research questions, this study aims to contribute valuable insights into the design, implementation, and optimization of CAs, as supportive tools in managing chronic health conditions. The findings presented in this paper seek to inform healthcare professionals and technology developers about the potential of these digital solutions to empower patients, improve health outcomes, and enhance the overall quality of life for different groups of people living with chronic diseases.

## Methods

This study employs a scoping review approach,^31^ which provides a comprehensive overview of the evidence on the chosen research topic. Our approach, therefore, is to give a relative breadth in our review rather than to focus on evidence around a single clinical or systematic question.

### Search strategy

The review followed the Preferred Reporting Items for Systematic Reviews and Meta-Analyses extension for Scoping Reviews (PRISMA-ScR) guidelines^32^ with the PRISMA-ScR checklist available as a Supplemental Material. We conducted a search of the literature using the electronic databases PubMed, ACM Digital Library, Scopus, and IEEE Xplore. These databases were chosen as they cover relevant aspects of health, technology, and interdisciplinary research and have also been used in other reviews covering similar topics. The main keywords used were “conversational agent” and “health” which were searched in titles and abstracts (Table 1).

**Table 1.** Search terms.

Term [conversational agent]		Term [health]
chatbot* OR “conversational agent*” OR “conversational system*” OR “conversational assistan*” OR “dialog* system*” OR “digital assistan*” OR “digital coach*” OR “relational agent*” OR “virtual agent*” OR “virtual assistan*” OR “virtual coach*” OR “automated messag*” OR “assistance technolog*” OR “smart speaker*” OR “automated assistan*” OR “intelligent personal assistan*"	AND	health* OR mhealth* OR uhealth* OR ehealth* OR healthcare OR illness* OR disease* OR treatment OR prevent* OR lifestyle OR wellbeing OR well-being OR medic* OR patien* OR caregiv*

### Study selection criteria

The review is based on literature dated between the beginning of January 2018 and the end of January 2023 published in English with full text available. We included articles on chronic disease management (e.g., treatment or monitoring) and prevention of specific chronic diseases. Articles that addressed general well-being were not included. Articles must provide some description of the theoretical basis, choice of the intervention components, or CA development process. We included both quantitative and qualitative studies, without any constraints on approach. Studies employing experimental designs that involve group comparisons must include details about the comparators used. Studies focusing only on technical aspects and design features of CAs (e.g., language models, systems, chatbot's personality) and studies using the Wizard of Oz method weren’t included.

#### Conversational agents

The review considers studies that involve interventions provided by a CA. This review does not include studies where CA focuses on video- and image-based diagnosis (e.g., skin cancer, gait, correct execution of exercises) and whose aim is screening before appointments, filling hospital forms, checking doctors’ availability, and giving diagnoses from the symptoms provided by the user.

#### Intervention

This review considers studies that evaluate intervention programs that include strategies to provide educational materials, help achieve health goals, and monitor health conditions. Interventions must not be intended for a hospital setting but can be tested in a laboratory environment. The intervention must be directly targeted toward the patients (e.g., not for emergency medical services, clinicians, or medical students).

#### Validity

This review considers studies that were tested on targeted user groups and have a report on the interventions’ impact on participants and/or participants’ experiences with the CA. Additionally, articles must have evaluation or validation of outcomes (e.g., acceptability, effectiveness) measured by reliable tools.

## Results

**Figure 1.**
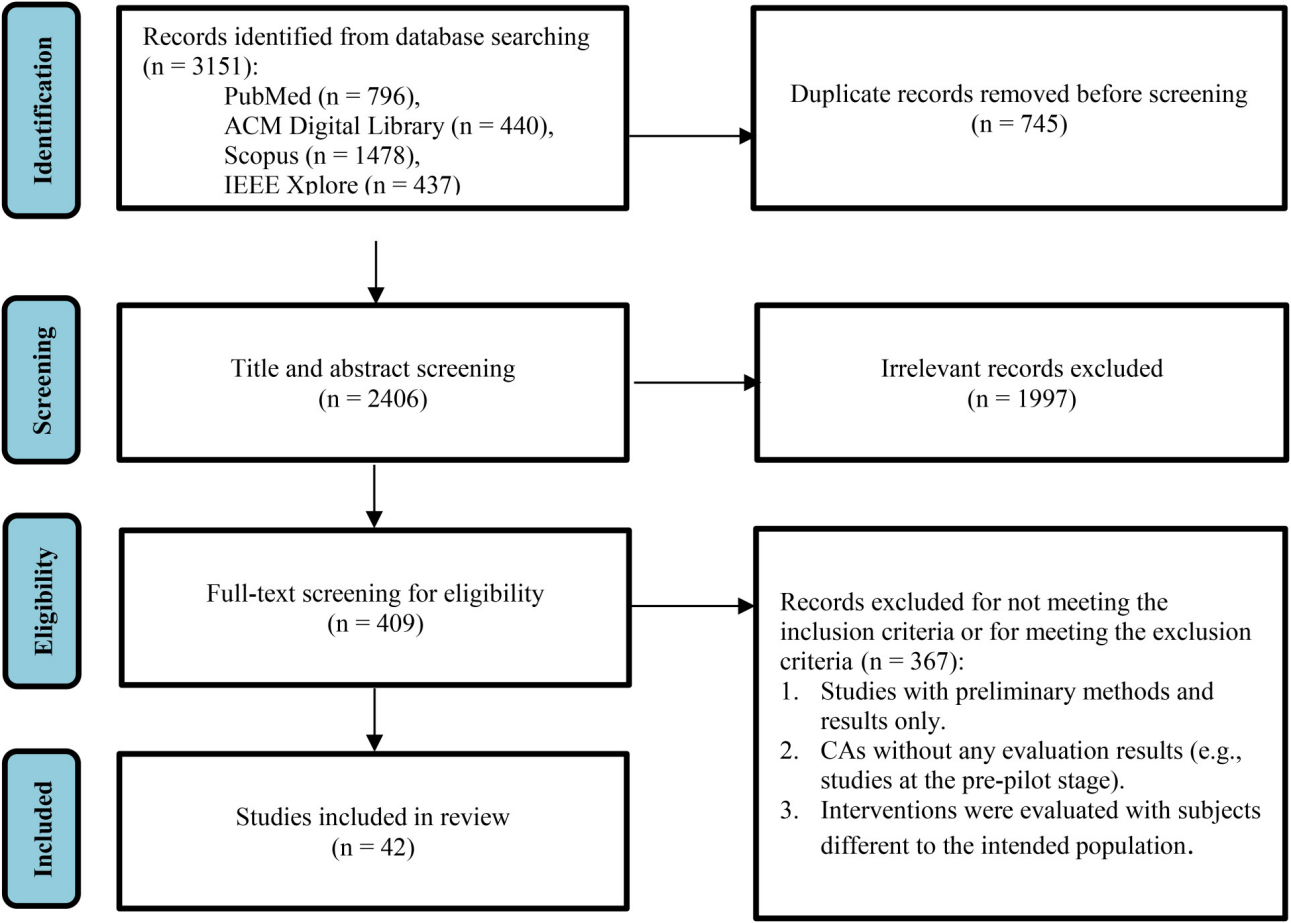
PRISMA-ScR^32^ flow diagram of included studies.

The literature search process from the four databases resulted in a total of 3151 research papers. The steps of the screening methodology are presented in Figure 1, along with the number of studies that were excluded in each step. The first step consisted of removing duplicate records of common studies that were found across all databases using the reference manager Zotero, which resulted in 2406 unique articles. All remaining unique studies were then considered in the initial title- and abstract-based screening. Following the proposed exclusion criteria described above, 409 articles remained. Full-text assessment of the remaining papers was then performed, resulting in a final list of 42 articles. The most relevant exclusion factor was validity, as many studies included only preliminary methods and results or presented CAs without any evaluation results (e.g., studies at the pre-pilot stage), or CAs that were evaluated with subjects different to the intended population.

### Characteristics of included studies

The full list of included studies is provided in Tables 2–7. Article publication dates ranged from 2018 to 2023. Eighteen studies were conducted in the USA,^8,10,33–48^ three each in Switzerland^49–51^ and China,^52–54^ two each in Australia,^55,56^ France,^57,58^ and the UK,^59,60^ and one each in Canada,^61^ Japan,^62^ Singapore,^63^ Saudi Arabia,^64^ South Africa,^65^ India,^66^ Germany,^67^ Italy,^68^ Norway,^69^ Spain,^70^ the Netherlands,^71^ and across Europe.^72^ The total number of participants ranged from 6 to 4737 and one study^69^ didn’t provide information on participant numbers. Participants aged between 5 and 86 years but not all articles provided information on age ranges.

**Table 2.** Health domains and targeted users’ characteristics.

Health domain	Indication	Phase in chronic disease continuum	Targeted users	References
Addictions and substance abuse	Smoking cessation	During	Veterans	^34^
	Substance use disorders	During	Alcohol users	^36^
			Adults positive for substance misuse	^33^
			Patients with methamphetamine use disorder	^52^
Asthma	Asthma	During	Children with asthma	^50^
Autoimmune disease	Celiac disease	During	Celiac patients	^64^
Cancer and obesity	Cancer and obesity	After-care	Overweight or obese cancer survivors	^41^
Cancer	Cancer	Prevention	Patients without cancer who are eligible for cancer genetic evaluation	^37^
	Cancer	After-care	Young people after cancer treatment	^39^
	Breast	Prevention	Women	^48^
		During	Women	^57,58^
	Multiple myeloma	During	Patients with multiple myeloma	^67^
Cardiovascular disease	Atrial fibrillation	During	People with atrial fibrillation	^8,40^
	Heart failure	During	Patients with heart failure	^35,61^
Chronic pain and mental health	Musculoskeletal pain and depression and/or anxiety	During	Adults with musculoskeletal condition and self-reported symptoms of depression and/or anxiety	^10,43^
Chronic pain	Chronic pain	During	People with chronic pain	^49^
	Neck/shoulder pain/stiffness and low back pain	During	Workers with neck/shoulder pain/stiffness and low back pain	^62^
Diabetes and mental health	Comorbid type 2 diabetes and depressive disorder	During	Patients with comorbid type 2 diabetes and depressive disorder	^70^
Diabetes	Diabetes	Prevention	General population	^63^
	Gestational	During	Pregnant women	^69^
	Type 1	During	Adolescents with type 1 diabetes	^71^
			Adolescents with type 1 diabetes and their parents	^47^
	Type 2	During	Patients with type 2 diabetes	^55,56,65,66^
	Types 1 and 2	During	Adults with diabetes	^68^
Functional bowel disorder	Irritable bowel syndrome	During	People with irritable bowel syndrome	^42^
Genetic condition	Sickle cell disease	During	Adults and young adults with sickle cell disease	^72^
Mental health	Depression and/or anxiety	During	Students	^38^
			Adolescents with depression and anxiety	^45^
	Complex, difficult-to-treat mental disorders	During	People with complex, difficult- to-treat mental disorders	^46^
	Depressive symptoms	During	Young adults with depressive symptoms	^53^
	Depression	During	Students with PHQ-9 score of 9 or higher^a^	^54^
	Mental health	During	Adults with mental health problems	^60^
Obesity	Obesity	During	Adolescents with obesity	^51^
			Children with obesity and their parents	^59^
	Weight management	During	Postpartum African American/ Black women	^44^

^a^ PHQ-9 score equal to or greater than nine was decided according to the average inclusion PHQ-9 score in previous depression trials.^54^

**Table 3.** Studies’ characteristics.

References	Participants’ characteristics					Duration	Study arms	Study approach	Measures
	Description	*N*	Mean age (range)	Gender/sex	Race				
^8^	With atrial fibrillation	151	71 (N/R)	N/R: female 49%	N/R	30 days	One	Qualitative, quantitative	AEI, satisfaction
^10^	With musculoskeletal condition	61	55 (18–83)	Sex: female 87%, male 12%, transgender 2%	White 90%	2 months	One	Quantitative	Feasibility, usability
^33^	With >1 on the CAGE-AID^a^	180	40 (18–65)	Sex: female 65%, male 35%	White 78%	8 weeks	Randomized Two: immediate and waitlist	Quantitative	Acceptability, feasibility, HO, TA
^34^	Smokers attempting to quit smoking	6	56.3 (37–76)	Sex: female 33%, male 67%	White 50% and AA 50%	14 days	One	Qualitative, quantitative	Acceptability, feasibility
^35^	Heart failure for 7 years on average	57	55 (>18)	Gender: female 35%, male 61%, missing 4%	Black 61%	90 days	Nonrandomized Two: smart speaker and visually animated and voice-enabled avatar	Quantitative	AEI
^36^	Alcohol users	51	28 (21–55)	Gender: female 32%, male 62%	White 42%, Black 24%	1 week	One	Qualitative, quantitative	AEI, acceptability, feasibility
^37^	Without cancer but with cancer family history	30	43.3 (25–60)	Sex: female 80%, male 19%	White 93%	10 minutes	One	Qualitative, quantitative	AEI
^38^	Students	74	22.9 (>18)	Gender: female 70%, male 28%, nonconforming 1%	Asian 50%, White 43%	2–4 weeks	Randomized Three: control (information), 2 weeks and 4 weeks intervention	Quantitative	AEI, HO, satisfaction
^39^	After cancer treatment with average 1.6 years post-completion of active cancer treatment	45	25 (19–29)	Gender: female 80%, male 20%	N/R	8 weeks	Randomized Two: immediate and waitlist	Qualitative, quantitative	Acceptability, AEI, feasibility, HO
^40^	With chronic atrial fibrillation	120	72.1 (N/R)	Gender/sex: female 52%	White 92.5%	30 days	Randomized Two: control (usual care) and intervention	Qualitative, quantitative	Acceptability, AEI, HL, HRQOL, MA
^41^	With mean BMI^b^ 32.9 kg/m^2^, 85.7% had stage 1 or 2 breast cancer	42	62.1 (N/R)	Sex: female 90%	N/R	4 weeks	Randomized Three: control (information), smart speaker and text-based intervention	Quantitative	Effectiveness
^42^	With gastrointestinal symptoms	121	32 (18–63)	Gender: female 75%, male 25%	White 76%	8 weeks	Randomized Two: immediate and waitlist	Quantitative	AEI, HO, HRQOL
^43^	With musculoskeletal condition	153	55 (18–86)	Sex: female 84%, male 16%	White 85%	2 months	Nonrandomized Three: control (usual care), usual care with in-person psychological counseling and with digital mental health intervention	Quantitative	HO
^44^	Postpartum AA/Black women with BMI^b^ 32.5 ± 4.3 kg/m^2^	136	27.8 (18–40)	N/R: female 100%	AA/Black 100%	12 weeks	Randomized Two: control (usual care) and intervention	Quantitative	Acceptability, AEI, HO, self-efficacy, SS
^45^	Had a new diagnosis of depression and anxiety in the past 3 months	18	14.7 (13–17)	Sex: female 88%, male 6%, missing 6%	White 88%	12 weeks	Randomized Two: immediate and waitlist	Qualitative, quantitative	Acceptability, AEI, feasibility, HO, safety^c^, usability
^46^	Enrolled in psychotherapy	73	37.3 (18–63)	Gender: female 89%, male 10%, genderqueer/androgynous 1%	N/R	4 weeks	One	Qualitative, quantitative	Feasibility, FDBT, HO, usability, usefulness
^47^	With T1D, disease duration 2–13 years, and their parents	26	N/R (10–15)	Gender: female 73%, male 27%	White 85%	3 months	One	Qualitative, quantitative	AEI, feasibility, satisfaction, TA, usability
^48^	Healthy females, 20% participants were identified as having high risk for breast cancer	30	22.5 (18–33)	Gender: female 100%	White 46.7%, Asian 36.7%	1 day	Randomized Three: control version of ECA, adaptive and non-adaptive ECA	Quantitative	Adoption, IMCB, HL, satisfaction
^49^	With chronic pain	102	43.77 (>18)	Gender: female 80%, male 20%	N/R	8 weeks	Randomized Two: intervention and motivational messages unrelated to chronic pain	Quantitative	Acceptance, AEI, HO, IMCB, TA
^50^	With an average 5.6 years since receiving the asthma diagnosis	49	12.04 (10–15)	Gender: female 33%, male 67%	N/R	30 days	One	Qualitative, quantitative	Acceptance, HL, TA, usability
^51^	With overweight or obesity	41	13.6 (10.9–16.9)	Sex: female 42%, male 58%	N/R	5.5 months	Randomized Two: control (usual care) and intervention	Quantitative	AEI, HO, usability
^52^	With methamphetamine use disorder, accumulated months of methamphetamine use 99.89	49	38.85 (18–55)	N/R	N/R	30–45 minutes	One	Quantitative	HO, IMCB, SP
^53^	Students with depressive symptoms, mean PHQ-9^d^ score 10.02	148	18.78 (17–21)	Gender: female 37%, male 63%	N/R	1 week	Randomized Three: an e-book, a general chatbot and mental health chatbot	Qualitative, quantitative	Acceptability, AEI, effectiveness, feasibility, HO, TA, usability
^54^	Students with a PHQ-9^d^ score of nine or higher	83	23.08 (19–28)	Gender: female 55%, male 45%	N/R	16 weeks	Randomized Two: bibliotherapy and chatbot	Qualitative, quantitative	AEI, HO, satisfaction, TA
^55^	T2D with mean HbA1c 7.3%, 56 mmol/mol	93	55.4 (N/R)	Gender/sex: female 47%	N/R	12 months	One	Qualitative, quantitative	Acceptability
^56^	T2D with the baseline mean HbA1c 7.3%, 56 mmol/mol	187	57 (>18)	Sex: female 41%, male 58%	N/R	12 months	Randomized Two: control (usual care) and intervention	Quantitative	AEI, effectiveness, HO, HRQOL, usability
^57^	With breast cancer in treatment or remission	142	42 (>18)	N/R: female 100%	N/R	1 day	Randomized Two: responses from chatbot and responses from physician	Quantitative	AEI, CCP
^58^	With breast cancer	4737	48 (13–65+)	N/R: female 89%, male 11%	N/R	1 year	One	Qualitative, quantitative	AEI, MA, usability
^59^	26 families with at least one child	34 children	8.4 (5–12)	Sex: female 50%	N/R	12 weeks	Randomized Two: control (no treatment) and intervention	Qualitative, quantitative	Acceptability, HO
^60^	With self-reported mental health problems	17	33.4 (22–67)	N/R: female 65%, male 35%	N/R	2 weeks	One	Qualitative, quantitative	AEI, HO
^61^	Patients with heart failure	20	57.8 (21–80)	Sex: female 45%, male 55%	N/R	4 weeks	One	Qualitative, quantitative	Acceptability, feasibility
^62^	With neck/shoulder pain/stiffness and low back pain	94	42 (20–64)	Sex: female 23%, male 77%	N/R	12 weeks	Randomized Two: control (usual care) and intervention	Quantitative	AEI, HO
^63^	With mean BMI^b^ 22.3 kg/m^2^	75	33.7 (>21)	Gender: male 38%	Chinese 80%	4 weeks	One	Quantitative	Acceptability, AEI, EIQOL, feasibility, HL, HO
^64^	Celiac patients	60	N/R	N/R	N/R	90 days	Randomized Two: control (no treatment) and intervention	Quantitative	AEI, feasibility
^65^	T2D (3708/4577 provided demographic information)	4577	51 (N/R)	Gender: female 55.7%, male 44%, other 0.3%	N/R	32 days	One	Qualitative, quantitative	Acceptability, adoption, appropriateness, feasibility, coverage^e^, HO
^66^	T2D	102	50.8 (N/R)	Gender: female 31%, male 69%	N/R	16 weeks	One	Quantitative	AEC, HO
^67^	Users (patients, caregivers, HCPs, etc.)	141	N/R	N/R	N/R	N/R	One	Quantitative	Usability, UX
^68^	T1D and T2D with mean duration of 10 years	13	30.08 (18–51)	N/R: female 77%	N/R	12 sessions of 10–20 minutes each	One	Qualitative, quantitative	AEI, HO, UX
^69^	610 dialogues, tool was available to all pregnant women in Norway	N/R	N/R	N/R	N/R	Open ended	One	Quantitative	AEI, UIN
^70^	T2D and depressive disorder	13	63.8 (44–83)	N/R: female 69%	N/R	9 months	One	Qualitative, quantitative	Acceptance, AEI, HO, IHR, MA, usability, usefulness
^71^	T1D	21	13.9 (12–18)	Gender: female 52%, male 48%	N/R	6–8 weeks	One	Qualitative, quantitative	HO, usability, UX
^72^	SCD, 64% affected by the most clinically severe SCD genotypes (Hb SS, HbSβ0)	33	37.37 (19–59)	Gender: female 67%, male 33%	N/R	1 day	One	Qualitative, quantitative	AEI, usability

*Note*. N/R: not reported; BMI: body mass index; HCP: healthcare professionals; SCD: patients with sickle cell disease; T1D: type 1 diabetes; T2D: type 2 diabetes; AA: African American; ECA: embodied conversational agents. AEC: association between program engagement and the change in HbA1c, FBG, and PPBG levels; AEI: adherence, engagement, and interaction; CCP: comparison of the average scores obtained by the chatbot and by the physicians for each individual; EIQOL: efficacy of the intervention with regard to the participants’ quality of life; FDBT: frequency of coping via dialectical behavior therapy skills; HL: health literacy; HO: health outcomes; HRQOL: health-related quality of life; IHR: impact on healthcare resources (number of medical appointments per month for each patient); IMCB: intention and motivation to change behavior; MA: medication adherence; SS: social support; SP: self-perception on the importance of drug abstinence and confidence in stopping the drug use; TA: therapeutic alliance; UIN: users’ informational needs; UX: user experience.

^a^ A cut-point of >1 on the CAGE-AID (cut down, annoyed, guilty, eye opener-adapted to include drugs) has a sensitivity of 70% and specificity of 85% for identifying individuals with substance use disorders.^33^

^b^ BMI between 25 and 29.9 kg/m^2^ falls within the overweight range, and BMI 30 kg/m^2^ or higher falls within the obese range. For the Asian and South Asian populations, overweight is when BMI is between 23 and 24.9 kg/m^2^, and obesity is when BMI is greater than 25 kg/m^2^.^73^

^c^ Parents were requested to provide information on any instances of their child being hospitalized or seeking medical attention for depression or anxiety-related issues in the preceding 2 weeks up to 1 month.

^d^ PHQ-9 assesses depression symptoms; scores of 5–9 predominantly represented patients with either no depression or subthreshold depression; patients with scores of 10 and higher are more likely to be diagnosed with depression.^74^

^e^ Number of people accessing the service and their demographic characteristics (age, gender, and language preferences).

**Table 4.** CAs’ purposes in the selected studies.

Purpose	References
Collect data	^35,51,52,61,64^
Deliver therapy and provide counseling	^10,33,36,38,41–43,45,49,51,52,54,60^
Encourage to think about quitting	^34^
Encourage physical activity	^41,42^
Provide feedback, summary, and suggestions	^35,36,41,44,52,55,70,71^
Provide information and education	^9,11,16,18–22,24,26,28–33,36,38–42,45–55^
Provide support	^10,38,43,45,51,54–56,68^
Send motivational and self-care messages	^61,62,66,71^
Send notifications and reminders	^10,50,58,59,62,70,71^
Symptom monitoring	^40,56^

**Table 5.** Factors impacting CA’s role in self-management.

References	CA personalization	Medical records	Human involvement
^10^	No	Use data in the study	Chat with expert
^35^	No	Use data in the study	Study nurse monitored alerts and evaluated participants’ stability
^36^	Provided individualized feedback about user's drinking habits, including information about risk factors and consequences	N/R	N/R
^37^	Provided more or less information based on user's preferences	Use data in the study, collect new data in the CA, and store it in the EHR	N/R
^38^	No	Possibility to integrate with existing EHR systems	N/R
^40^	The content was tailored for individual use by using the user's name and appropriate time context	Use data in the study	N/R
^41^	The intervention intensity and frequency depended on participant's motivation to seek coaching. MyCoach used a reinforced recommendation system to learn about participant behavior to maximize rewards. The unidirectional coach customized messages based on individual factors like schedule, sensors, preferences, and progress.	Use data in the study	N/R
^43^	No	Use data in the study	Chat with expert
^44^	Provided personalized feedback on weight changes specific to the data entered	N/R	N/R
^45^	Tailored the conversation to the present situation to help the adolescent develop emotion regulation skills in the context of their everyday life, offered, and guided the user through cognitive behavioral therapy-based psychoeducation and tools, tailored to the reported need	Collect and record data	N/R
^49^	Personalized text messages	N/R	N/R
^50^	Asked about user's emotional state and provided personalized feedback based on their answers	N/R	Communicate with HCP, patients, and family members
^51^	No	N/R	Monitor, include standardized counseling and ability to chat through app, patients were able to chat with each other
^52^	No	N/R	Psychotherapists or social workers can add new subjects and refer to patients’ assessment and treatment records
^54^	No	N/R	Professionals will intervene by telephone when the participants report a psychological emergency
^55^	Delivered personalized support, monitoring, and motivational coaching. Algorithms were tailored according to the clinical targets and recommendations provided by each participant's general practitioner	N/R	N/R
^56^	Personalized support, monitoring, and motivational coaching	Use data in the study	N/R
^57^	Personalized text messages	N/R	N/R
^58^	Personalized text messages	N/R	N/R
^61^	Generated an automated self-care message based on the data inputted and the patient's medical history	Use and store data in the app	N/R
^62^	Offered tailored replies depending on responses	N/R	N/R
^66^	Provided educational, behavioral, and motivational messaging specific to the data entered and in the context of the patient's previous clinical, lifestyle, and behavioral data	Use and store data in the app	Voice calls and chat with expert
^67^	No	N/R	No, but has a function to search for local German support groups
^70^	No	N/R	HCP could monitor patients
^71^	Provided personalized feedback, rewarded goal-aligned behaviors, and tailored medical content and coaching messages through shared decision-making with healthcare professionals	Integrate the findings in existing patient web portals for T1D	Message exchange between patients, their peers and their caregivers
^72^	Addressed participants’ accountability by referring to earlier data entered, tasks, or activities performed	N/R	N/R

*Note*. The table includes only studies that contain at least one of the discussed components. N/R: not reported; T1D: type 2 diabetes; HCP: healthcare professionals.

**Table 6.** Underpinning frameworks in the selected studies.

Health domain	Underpinning framework	References
Addictions and substance abuse	US Clinical Practice Guidelines	^34^
	Brief motivational interviewing, technology acceptance model	^36^
	Cognitive behavioral therapy, dialectical behavior therapy, motivational interviewing, mindfulness training	^33^
	Cognitive behavioral therapy, motivational enhancement therapy, mindfulness-based relapse prevention	^52^
Asthma	Behavior change techniques, experiential learning theory, self-determination theory, theory of planned behavior	^50^
Autoimmune diseases	Chronic-disease extended model	^64^
Cancer	N/R	^57,58,67^
	Cognitive theory of multimedia learning, adult learning theory	^37^
	Stress and coping theory, broaden-and-build theory of positive emotions	^39^
	Heuristic-systematic model of information processing	^48^
Cancer and obesity	N/R	^41^
Cardiovascular diseases	N/R	^35,40,61^
	Chronic care model	^8^
Chronic pain	N/R	^62^
	Cognitive behavioral therapy	^49^
Chronic pain and mental health	Cognitive behavioral therapy, dialectical behavior therapy, deep breathing techniques, motivational interviewing, mindfulness training, sleep meditations	^10^
	Cognitive behavioral therapy, motivational interviewing, mindfulness training	^43^
Diabetes	N/R	^55,65,69^
	Social cognitive theory, self-determination theory	^47^
	Behavior change techniques, transtheoretical model, social cognitive theory, gamification	^56^
	Distance learning-based stress management techniques, COM-B model	^63^
	Digital persuasion model, American Association of Diabetes Educators framework	^66^
	Obesity-related behavioral intervention trials framework, mindfulness-based cognitive therapy, American Association of Diabetes Educators framework	^68^
	Behavior change techniques, goal setting theory, persuasive system design model, self-determination theory	^71^
Diabetes and mental	N/R	^70^
Functional bowel disorder	Cognitive behavioral therapy	^42^
Genetic condition	World Health Organization’ handbooks on how to implement text-based mHealth interventions	^72^
Mental health	Acceptance and commitment therapy, cognitive behavioral therapy, emotionally focused therapy, interpersonal psychotherapy, mindfulness-based cognitive therapy, motivational interviewing, solution-focused brief therapy, self-compassion therapy, transtheoretical model	^38^
	Cognitive behavioral therapy, dialectical behavior therapy, interpersonal psychotherapy for adolescents	^45^
	Dialectical behavior therapy	^46^
	Cognitive behavioral therapy	^53^
	Bibliotherapy, cognitive behavioral therapy	^54^
	Method of levels, perceptual control theory	^60^
Obesity	N/R	^44,59^
	Behavior change techniques	^51^

*Note*. N/R: not reported.

**Table 7.** CAs’ technical characteristics in the selected studies.

Delivery channel	User input modality	Agent output modality	References
Amazon Alexa	Free speech	Speech, VA	^59,67^
Amazon Alexa and mobile phone	Free speech	Speech, VA	^41^
	No	Text, UC	
Amazon Alexa and tablet-based app	Free speech	Speech, VA	^35^
	Free speech	Text, speech, ECA	
Digital platform	Fixed, (sometimes free) text	Text	^37^
	Fixed, free text	Text	^69^
Gamification platform	No	Text, UC	^71^
Messaging app	Fixed text	Text, image	^63^
	Fixed text	Text	^70,72^
	Fixed text	Text and audio	^65^
	Fixed, free text	Text	^38,68^
	Free text	Text	^39^
	Free text, speech	Text	^54^
	N/R	Text	^64^
	N/R	Text, image, and voice	^53^
Mobile app	Fixed text	Speech, ECA	^8^
	Fixed text	Speech, RA	^40^
	Fixed text	Text	^46,51^
	Fixed text, speech	Speech, ECA	^55,56^
	Fixed text	Text, media	^62^
	Fixed, (sometimes free) text	Text, media	^49^
	Fixed, (sometimes free) text	Text	^50^
	Fixed, free text	Text	^10,43^
	Free speech	Speech, VA	^61^
	N/R	Text	^42,66^
	N/R	Text, RA	^33,45^
	No	Text, UC	^44^
Tablet-based app	Fixed text	Speech, ECA	^52^
	N/R	Speech, ECA	^34^
Web app	Fixed text	Speech, ECA	^48^
	Fixed text	Speech, RA	^47^
	Fixed, free text, speech	Speech, ECA	^36^
	Free text	Text, RA	^60^
Web, mobile, and messaging app	Fixed, free text	Text	^58^
	Free text	Text	^57^

*Note*. ECA: embodied conversational agents; RA: relational agent; VA: voice assistant; UC: unidirectional coach.

### What are the health domains and the characteristics of the users, targeted for CA interventions?

When creating technology for a specific health domain, it is essential to consider symptoms, treatment requirements, and challenges associated with that domain that people may encounter at different stages. Moreover, understanding users’ characteristics, such as demographics, medical history, cultural background, goals, and preferences, allows the content and interaction strategies to be tailored. This enhances engagement, adherence, and overall health outcomes.^75,76^ Table 2 shows information about the health domains addressed by the CAs in the selected articles, providing the context of the disease, including phase, e.g., prevention, after-care or during (between onset and end of the disease), as well as information about the target users.

#### Health domains

Among the various types of chronic conditions, different types of diabetes (type 1, type 2, gestational diabetes, and prediabetes)^47,55,56,63,65,66,68,69,71^ and mental health issues (depression, depressive symptoms, anxiety, bipolar disorder, and other complex, difficult-to-treat mental disorders)^38,45,46,53,54,60^ received the most attention. Other types of chronic conditions included six studies on cancer (multiple myeloma, breast cancer, cancer genetic evaluation, and after cancer treatment),^37,39,48,57,58,67^ four studies on addictions and substance abuse (smoking cessation, alcohol and methamphetamine use disorder, and substance misuse),^33,34,36,52^ four studies on cardiovascular disease (heart failure and atrial fibrillation),^8,35,40,61^ three studies on obesity,^44,51,59^ two studies on chronic pain,^49,62^ and one study each on asthma,^50^ autoimmune (celiac disease),^64^ genetic condition (sickle cell disease),^72^ and functional bowel disorder (irritable bowel syndrome).^42^ Additionally, there were four studies focused on comorbid diseases: two on musculoskeletal conditions and mental health,^10,43^ one on type 2 diabetes and depressive disorder,^70^ and another one on obesity and cancer.^40^

Most of the systems were aimed at supporting people who already have chronic diseases (37 studies). There were only three studies that highlighted their focus on prevention: one study focused on patients without cancer who were eligible for cancer genetic evaluation,^37^ one on communicating breast cancer risk and the recommended medical guidelines to healthy women,^48^ and another on promoting healthy lifestyle behavior changes with the focus on diabetes and prediabetes knowledge in the general population.^63^ Two studies focused on after-disease care: one study concentrated on overweight or obese cancer survivors^41^ and another on young people after cancer treatment.^39^

#### Targeted users’ characteristics

In the selected studies, people with chronic conditions, or the risk of acquiring one were the most common final targeted interaction recipients. Only three studies targeted the interaction of patient–parent dyads.^47,50,59^

The age groups of target users varied. There were six studies focused on children and teenagers.^45,47,50,51,59,71^ Four studies targeted young adults.^38,39,53,54^ The remaining 32 studies were for different groups of adults.

There was no information about the duration of illness, medication usage, number and type of comorbidities, employment and marital status or any other socio-demographic information of target users that might be important for intervention development. Only a few studies were targeted recipients additionally specified; two studies targeted university students,^38,54^ one study focused on veterans,^34^ one specifically targeted African American/Black women,^44^ and one had a focus on workers.^62^

### What are the characteristics of the CA studies?

When conducting studies, it can be meaningful to test interventions with multiple study arms to compare them, evaluate their efficacy and inform decision-making. Conducting studies with participants representative of the target group enhances applicability and generalizability that improves the validity and relevance of findings for the interventions’ intended users. Knowing the intervention duration can be important for the understanding of the impact and effectiveness of the interventions. Moreover, chosen measure types guide the evaluation of health apps, ensuring evidence-based interventions and objective outcome assessment. Thus, effectiveness, a fundamental aspect of intervention assessment, can be measured through various outcome indicators chosen by researchers, such as symptom reduction, changes in health behavior and health indicators (e.g., blood pressure, glucose levels), improvements in quality of life, and others. These measures serve as quantifiable evidence of an intervention's success in achieving its intended goals. The characteristics of the studies are summarized in Table 3. If there were multiple race groups participating in the study, we mention only those above 20%. We reported gender and sex as explicitly stated in the studies. If the totals do not add up to 100, it means this information was not provided in the studies.

#### Participants

The age of the research participants corresponded to the targeted users’ descriptions since this was a criteria for inclusion in the review. There were six studies focused on children and teenagers aged from 5 years to 18 years old.^45,47,50,51,59,71^ Four studies targeted young adults, 17–29 years old.^38,39,53,54^ One study had a wide range age of targeted users and participants from 13 to 65+ years old.^58^ The remaining 31 studies involved different groups of adults. Three studies didn’t provide any information about participants,^64,67,69^ except that they corresponded to the target users, e.g., already had the health condition or risk of acquiring the disease. Moreover, one of these studies, Baertsch et al.,^67^ besides patients, had healthcare professionals and caregivers among the users since the program was advertised and had open access.

Apart from age, gender, or sex was frequently mentioned in the articles as a demographic characteristic of the participants. There were 17 articles that used the term “gender,” 12 that used the term “sex,” 2 that used both terms, and 11 did not specify. Unfortunately, it is often impossible to determine exactly what researchers meant when they used a particular term. Furthermore, some of the articles provided additional information on specific health indicators, such as body mass index (BMI) or disease duration, employment and marital statuses, race, and ethnicity. Having a more detailed description provides valuable insights for understanding the diverse demographics and health profiles of the participants, allowing conducting in-depth analysis of the interventions’ effectiveness and relevance across different populations.

#### Duration

There were two studies with unspecified duration.^67,68^ Of those with specified domains, there was one study^69^ that collected data over 20 weeks (there were two periods: 8 and 12 weeks), though it is not clear for how long and how many times each user engaged with the chatbot since the data were anonymous and the identification of users was not permitted. Five studies took only 1 day to finish the planned intervention.^37,48,52,57,72^ Two studies each were for 1 week and 2 weeks, nine studies were about 1 month long, seven studies had a 2 month duration, and eight studies had a 3 month duration. Several studies lasted more than 3 months: two studies for about 4 months,^54,66^ one study for 5.5 months,^51^ one study for 9 months,^70^ and 3 studies for 1 year.^55,56,58^

#### Study arms

Most of the studies were single-armed (22/42). There were 15 two-arm studies, 11 of which compared intervention and control groups (see Table 3), including five that compared intervention and usual care,^40,44,51,56,62^ four that compared immediate intervention and waitlist control groups,^33,39,42,45^ and two compared intervention and control groups with no treatment.^59,64^ Another setup encountered is a comparison of two alternative interventions: responses from chatbot versus responses from physicians,^57^ interaction with a chatbot concerning pain management versus reception motivational messages unrelated to chronic pain,^49^ bibliotherapy versus chatbot interaction,^54^ and smart speaker versus visually animated and voice-enabled avatar interaction.^35^ There were also five three-arm studies, that compared an e-book, a general chatbot, and mental health chatbot^53^; control group that only received a link to a book, 2 and 4 weeks intervention groups^38^; control version, adaptive, and non-adaptive embodied conversational agent (ECA)^48^; control group that received printed written information, smart speaker, and text-based intervention^41^; and usual orthopedic care without any specific mental health intervention, usual orthopedic care with in-person psychological counseling and usual orthopedic care with digital mental health intervention.^43^

#### Measures

A diverse range of measures was used to assess the interventions in the selected studies. One of the most common measures were health outcomes (23/42) which were measured by physical assessments like BMI, glycated hemoglobin level, blood pressure, and heart rate,^44,51,56,59,66,70^ and questionnaires, such as Generalized Anxiety Disorder-7, Patient Health Questionnaire-9, International Physical Activity Questionnaire, Pittsburgh Sleep Quality Index, and Perceived Stress Scale.^33,38,39,42–46,49,51–54,56,59,60,62,63,65,68,70–72^ All the studies demonstrated improvement, but not always statistically significant.

The same metrics are often used to evaluate engagement, adherence, and interaction with apps and CAs, e.g., number, lengths and context of messages sent, and this can create challenges in distinguishing the specific aspect being assessed. Among the selected studies, 26 evaluated chats with CAs and activity in the apps (we called this measure Adherence, Engagement, and Interaction in Table 3), which was measured by a variety of methods, including analysis of interview transcripts,^8^ chat transcripts and metrics, e.g., number and length of messages,^35,37–40,45,49,51,53,56,58,60,62–64,69,70^ app usage, e.g., number of accesses to the app and videos watched,^42,44,45,56,70^ different questionnaires, e.g., Godspeed for assessing human-like traits of CA,^36^ the Usefulness Scale for Patient Information Material for evaluating perceived usefulness^72^ and the User Engagement Scale–Short Form questionnaire for measuring self-reported user engagement,^68^ and other surveys to evaluate various aspects, e.g., the level of confidence, attitude, and perceived quality of the answers.^39,47,54,57,58^ Many metrics based on chat and app usage provided quantitative values that were evaluated relative to each other, such as the number of messages sent weekly. The results indicated that participants found the CAs to be useful, reported overall positive attitudes, and showed high engagement and adherence rates. All other 26 measurements identified were used in fewer than 13 articles.

#### Effectiveness

Most of the studies didn’t talk explicitly about the effectiveness. It can be assumed that effectiveness of the intervention is determined by the results of the selected measures, as noted in the previous paragraph, such as health outcomes, usability, and engagement. However, there are several articles that mentioned why they didn’t provide information about effectiveness; some of the studies weren’t designed to test the effectiveness,^63^ other couldn’t establish it for different reasons, e.g., the small sample size and/or insufficient intervention duration.^45,46,51,59,60^

### Do CAs address continuum of care and patient work concepts for self-management? Do CAs adapt to the changing needs of users?

CAs play a multifaceted role in chronic disease management, addressing the continuum of care by providing, for example, education, remote monitoring, and seamless health records integration that can enable access to comprehensive patient data that contributes to creating personalized and tailored interventions. CAs contribute to patient work through symptom tracking and coaching, empowering patients and caregivers to self-manage their health. Moreover, human involvement, e.g., relatives, peers, and healthcare professionals, in chronic condition management can provide social support and motivation for patients, enhancing adherence to the interventions and overall well-being.^77,78^ Furthermore, the inclusion of medical records in health applications can facilitate the development of personalized interventions, resulting in enhanced health outcomes, greater user engagement, and heightened satisfaction.

The purpose of the reviewed CAs are presented in Table 4 and the factors impacting CA's role in self-management are shown in Table 5 for any study that mentioned the CA's factors.

#### CA purposes

In the selected studies, CAs had a diverse range of purposes. The most common ones were providing information and education (33/42), delivering cognitive therapy (12), and providing mental health and emotional support (11) (see Table 4). All other purposes, such as providing feedback, collecting data, sending reminders, and motivational messages and monitoring symptoms, were mentioned fewer than six times.

#### CA personalization

Twenty-five CAs didn’t adopt message content to users’ characteristics at all (see Table 5). Five studies mentioned that CAs sent “personalized text messages” and “tailored replies depending on users’ responses” without further explanation.^49,56–58,62^ In one study participants can choose to receive more or less details on the topics based on their preferences.^37^ Based on participants’ goals, habits, and data entered during a conversation with CA, four CAs provided personalized feedback,^36,44,50,71^ and four CAs sent personalized educational, motivational, and coaching messages.^41,55,61,66^ One CA tailored the conversation to the user's present situation and to the reported need at that moment,^45^ one CA referred to earlier data entered, tasks, or activities performed and added user's name^72^ and another CA added user's name and appropriate time context to the messages, e.g., “Good Afternoon John.”^40^

#### Medical records

We have only considered the usage of medical records in conjunction with the CAs. For example, cases when medical records were used for recruiting participants are not demonstrated in Table 5. Thirty studies didn’t mention any connection to patients’ medical records (see Table 5). Nine studies used medical records data for the studies.^10,35,37,40,41,43,56,61,66^ One study recorded information after the study to participants’ electronic health record (EHR)^37^ and one study used participants’ medical charts to confirm any additional mental health diagnoses and record their initial treatment plan.^45^ Moreover, one study aimed to integrate the findings in existing patient web portals that support the care of patients with type 1 diabetes,^71^ and one study noted the possibility of integrating the chatbot's administration panel into the existing EHR system.^38^ One study collected medical history and stored it in the app when the patient was onboarded to the program^61^ and one study stored personal health record files with additional diabetes-related information (e.g., laboratory reports and details of treating physician) that users logged in the mobile application.^66^ Among these 12 studies, there were three on diabetes,^56,66,71^ three on cardiovascular conditions,^35,40,61^ two on mental health,^38,45^ two on comorbid chronic pain and mental health,^10,43^ one on cancer,^37^ and one on comorbid cancer and obesity.^41^

#### Human involvement

Despite the evidence that human involvement, e.g., family members, peers, and healthcare professionals, in the management of chronic conditions can offer patients social support and motivation,^77,78^ in 31 studies there was no human involvement in the intervention groups (see Table 5). Four studies allowed the participants to communicate with health experts, two studies only through chat,^10,43^ one through chat and face-to-face interaction^50^ and one through chat and voice calls.^66^ In three studies, healthcare professionals monitor participants’ engagement and results.^35,52,70^ In one study, healthcare professionals could intervene by telephone (Mental Assistance Hotline) when the participants reported that they needed emergency psychological assistance^54^ and in another study, the app alerted healthcare professionals in case of a lack of chat interaction during more than 2 days and four standardized counseling on-site visits, two via phone and 10 minutes of interaction through chat app was included.^51^ Family members were included in one study by Kowatsch et al.,^50^ CA could send SMS text messages to them, and healthcare professionals had a possibility to communicate with family members through the app. In Stasinaki et al.^51^ patients were able to chat with each other and in Klaassen et al.^71^ there was a message exchange between patients, their peers, and their caregivers. In two of the studies without human involvement the CA motivated users to join online peer-support patient communities or request health coaching from a human^72^ or search for local support groups.^67^

The targeted health domains for the studies with human involvement were diabetes,^66,71^ comorbid chronic pain and mental health,^10,43^ mental health,^54^ comorbid diabetes and mental health,^70^ asthma,^50^ cardiovascular conditions,^35^ addictions and substance abuse,^52^ cancer,^67^ and obesity.^51^

### What are the CAs’ characteristics? How do different types of CAs map with patients’ profiles and health domains?

Choosing or designing CAs suitable to target users requires an understanding of their characteristics, such as type of CA, input and output modalities, as these influence the user experience and engagement. The input modality refers to how users interact with the CA, such as through text, speech, or by choosing from the responses menu, while the output modality relates to the CA's responses, whether in text, speech, or visual forms. Moreover, different types of CAs have unique capabilities and advantages. For instance, relational agents focus on building and maintaining long-term, social–emotional relationships with users, fostering a sense of trust and empathy^79^; ECAs have the same properties as humans in face-to-face conversation, including producing and responding to verbal and nonverbal communication^80^; and task-oriented dialogue agents are designed to perform a specific function or deliver a particular service, such as providing educational tutorials. These agents are different from general-purpose CAs, which are more versatile and capable of handling a wide range of tasks and conversations.^81^

Theoretical frameworks are essential when developing health interventions and applications as they provide a structured and evidence-based foundation, guiding the design, implementation, and evaluation process to ensure effectiveness and alignment with established health behavior principles.

Underpinning frameworks are presented in Table 6 and CAs’ technical characteristics are in Table 7.

#### Theoretical frameworks

One-third of all the studies (14/42) did not mention any theoretical base on which the interventions and the application design were based (see Table 6). The rest of the studies had a lot of variety in the theories, therapies, techniques, and frameworks that were considered when developing the studies. A total of 40 different theories were mentioned, with cognitive behavioral therapy, mindfulness (includes training and all mindfulness-based therapy and techniques), motivational interviewing (includes brief motivational interviewing), dialectical behavior therapy, behavior change techniques, and self-determination theory being referenced most frequently (10, 6, 5, 4, 3, and 3 times, respectively), with the remaining 33 theories having no more than two mentions each. All the frameworks can be categorized into several domains: psychotherapy and counseling theories (acceptance and commitment therapy, cognitive behavioral therapy, dialectical behavior therapy, emotionally focused therapy, interpersonal psychotherapy, motivational enhancement therapy, solution-focused brief therapy), health behavior change theories (behavior change techniques, goal setting theory, self-determination theory, social cognitive theory, stress and coping theory, theory of planned behavior, transtheoretical model), educational and learning theories (adult learning theory, cognitive theory of multimedia learning, experiential learning theory), healthcare models and guidelines (American Association of Diabetes Educators (AADE) framework, chronic care model, chronic-disease extended model, obesity-related behavioral intervention trials framework, US Clinical Practice Guidelines, World Health Organization's handbooks on how to implement text-based mHealth interventions), mind-body techniques (mindfulness techniques, sleep meditations, deep breathing techniques), technology and digital health frameworks (digital persuasion model, persuasive system design model, technology acceptance model).

#### CAs’ characteristics

The majority of CAs in the reviewed studies were text-based (28 out of 42) (see Table 7). Seven CAs only allowed users to choose an answer from the options provided^46,51,62,63,65,70,72^ and three CAs had several free text questions, e.g., provide the user's name.^37,49,50^ Among these CAs, four sent media, like audio lessons and pictures with exercises, to users.^49,62,63,65^ In three studies, participants could type their responses freely^39,57,60^ and, in Liu et al.,^54^ participants could also send voice messages to the chatbot. In six CAs, users could both choose responses from a menu and send free text messages or choose from the responses menu.^10,38,43,58,68,69^ Six studies didn’t specify users’ input format.^33,42,45,53,64,66^ There were three studies with unidirectional coaches where the CAs sent messages to the participant, but the participant could not communicate with the agent.^41,44,71^ Four studies based their intervention on Amazon's Alexa,^35,59,61,67^ including one that compared Amazon's Alexa with a visually animated and voice-enabled avatar.^35^ Additionally, one study compared unidirectional coach with Amazon voice assistant.^41^ Ten studies used an ECA to provide interventions,^8,34–36,40,47,48,52,55,56^ the users’ input modes varied: most systems allowed interaction with the ECA only with predefined answer options,^8,48,52^ while others allowed voice and/or free text messages.^36,55,56^

Among the selected studies, there were five with relational agents designed to build and maintain long-term emotional relationships with their users.^33,40,45,47,60^ Three of them were text-based for mental health and addictions and substance abuse^33,45,60^ and two were ECA targeting diabetes and cardiovascular issues.^40,47^

## Discussion

Advancements in technologies have facilitated a plethora of mobile apps specifically designed to support and improve health habits that give patients more control and a sense of agency, empowering them to take charge of their health.^82^ Existing digital technologies for patients with chronic conditions face challenges in adapting to changing health needs and goals, requiring diverse information and recommendations for specific subgroups.^13^ Implementing adaptable digital systems can enable sustained app usage, supporting individuals in effectively managing their health and reducing disruption caused by switching between different apps.

Relevant studies were obtained from the PubMed, ACM Digital Library, Scopus, and IEEE Xplore databases. The methodology adopted in this review aligned well with the PRISMA-ScR guidelines and checklist^32^ process, see the Supplemental Material for further details. The review included 42 studies reporting interventions delivered by CAs, targeting chronic diseases. Diabetes, mental health, and cancer are the diseases most commonly targeted by CA interventions, as opposed to other chronic conditions, such as autoimmune, genetic conditions, functional bowel disorders, and asthma. The review shows that current CAs address a wide variety of chronic diseases. Although it provides valuable insights, the extensive range of conditions complicates the ability to compare them within and between conditions. To address this, future research should focus on evaluating CAs specifically tailored to particular chronic diseases.

The overall trends can be summarized as follows:

### Focus on stable chronic condition management

The analysis reveals that the majority of studies have primarily targeted individuals who already have chronic diseases. There seems to be a disproportionate emphasis on stable chronic condition management rather than on other stages of the disease where people might have other needs, for example, newly diagnosed patients or patients after crises. Only one study mentioned it was designed to address current and future patients’ needs.^58^ This finding highlights a potential gap in leveraging digital interventions that adapt to the changing needs of people with chronic diseases, which could significantly impact the overall burden of these conditions on healthcare systems.

### Limited representation of specific demographics

The research highlights the lack of studies focusing on certain demographic groups, including children/teenagers and older adults, which aligns with previous reviews.^22^ While interventions for adults aged 18 and above are relatively abundant, there is a noticeable gap in providing support for younger and older populations. Understanding the specific challenges and requirements of these groups is crucial for designing age-appropriate and inclusive digital health solutions.

### Incomplete participant information

For this review, we selected studies featuring participants who matched the targeted user group. Yet, there is often limited information about the participants beyond basic demographics such as age and gender or sex. Since studies sometimes did not explicitly report all values beyond the primary gender or sex and the terms “gender” and “sex” can be used interchangeably, with “gender” increasingly used to describe biological variations traditionally assigned to “sex,”^83^ comparing these studies becomes challenging. Marital status, ethnicity, and other socio-economic features are often missing. Moreover, studies failed to provide a thorough comprehension of participants’ needs, aligning with the previous reviews.^22,28^ This lack of comprehensive participant information might hinder a thorough understanding of the interventions’ applicability and effectiveness across diverse populations.

### Importance of human involvement

While CAs offer promising directions, the limited provision for human interaction within these systems is identified as a potential drawback. Human support from healthcare professionals, family members, and peers with similar health problems can provide emotional encouragement, motivation, and vital insights that are essential for successful long-term management of chronic conditions.^77,78^

### Limited integration of medical records

The review indicates that limited use of medical records by the CAs potentially hinder smooth communication and collaboration between individuals and healthcare professionals. Moreover, medical records in health apps can enable the creation of personalized and tailored interventions that lead to improved health outcomes and increased user engagement and satisfaction.

### Insufficient information on theoretical framework

Several studies didn’t provide information about the theoretical framework guiding the design and implementation of interventions. This omission raises concerns about the basis for the interventions and highlights the importance of incorporating robust theoretical foundations in future research and development.

### Lack of unified evaluation measures

In accordance with prior reviews, we also found that the evaluation measures used to assess the effectiveness of the interventions and their impact on chronic conditions varied widely across studies.^15,28^ This lack of standardization makes it challenging to compare and generalize findings.

### Limited use of technologies and user information

There is potential for CAs to assist people in actively managing their conditions instead of passively consuming information. However, most current CAs have dialogue management systems that do not consider user preferences, goals, or history of interaction with the system. To better serve user needs, the design of these agents should evolve. The linguistic data generated by users during interaction with CAs holds the capacity to provide insights into users’ emotional and physical states. These data can help people with self-management and provide valuable information for the patients’ support team. A scoping review on psychology-oriented ECAs revealed that most agents were still in their initial phases of development and evaluation,^23^ aligning with our results. A systematic review on health-related CAs with unconstrained language input capabilities found limited use of agent-based systems capable of handling complex dialogues and allowing users to lead conversation.^28^ The review found only one study evaluated such systems in health contexts, but agents weren't designed for health-related queries. The authors indicated the requirement for large training datasets as a major drawback, potentially slowing their adoption in health applications.

Another factor to consider is that, at the time of writing, advances in the application of large language models offer significant opportunities in various fields, including healthcare. However, this has not yet been reflected in the available literature. It will be interesting to observe how this technological progress could potentially address the highlighted issues.

## Strengths and limitations

The main strength of this review is its novelty. To the best of our knowledge, this is the first scoping review addressing different types of CAs in chronic disease management with no constraints on demographics. Nevertheless, it is essential to acknowledge that this analysis has its limitations. First, given that the research question is very broad, a detailed review of the use of CAs in different chronic diseases was not possible. Second, the review was exclusively focused on text and voice CAs, omitting those utilizing images and video. Third, we included articles in English with full text available only and the search in other databases, such as CINAHL and Web of Science, was not conducted. Fourth, our analysis only incorporated features, e.g., theoretical frameworks, explicitly mentioned in the articles. Therefore, certain characteristics may not be fully represented in our review, potentially limiting the generalizability of the conclusions due to potential bias.

## Conclusions

This paper highlights the current state of CAs for chronic condition management and raises important considerations for future research and development in this field. The findings emphasize the health application requirements to adapt to the changing needs during the course of illness that varies for each patient and changes over time, customize interventions based on specific user subgroups, and improve the reporting of study participant characteristics to enhance the applicability of the findings. It also underscores the importance of incorporating human support and medical records integration within digital health solutions to provide more effective care for individuals living with chronic conditions. Additionally, studies should clearly articulate the theoretical frameworks guiding their interventions, and efforts should be made to standardize evaluation measures to facilitate meaningful comparisons between studies. Future research could more closely explore studies on each particular disease to gain a deeper understanding of CAs’ use and potential in disease management.

## Supplemental Material

sj-docx-1-dhj-10.1177_20552076241277693 - Supplemental material for Exploring the characteristics of conversational agents in chronic disease management interventions: A scoping review Supplemental material, sj-docx-1-dhj-10.1177_20552076241277693 for Exploring the characteristics of conversational agents in chronic disease management interventions: A scoping review by Ekaterina Uetova, Lucy Hederman, Robert Ross and Dympna O’Sullivan in DIGITAL HEALTH
